# Supplementary material for: Empagliflozin Treatment Attenuates Hepatic Steatosis by Promoting White Adipose Expansion in Obese TallyHo Mice
Source: Int J Mol Sci. 2022 May 18;23(10):5675. doi: 10.3390/ijms23105675 (PMC9147974; doi:10.3390/ijms23105675)
Supplement: Supplementary file 1 [file ijms-23-05675-s001.zip › Figure S3.pdf]

Supplemental Figure S3

A

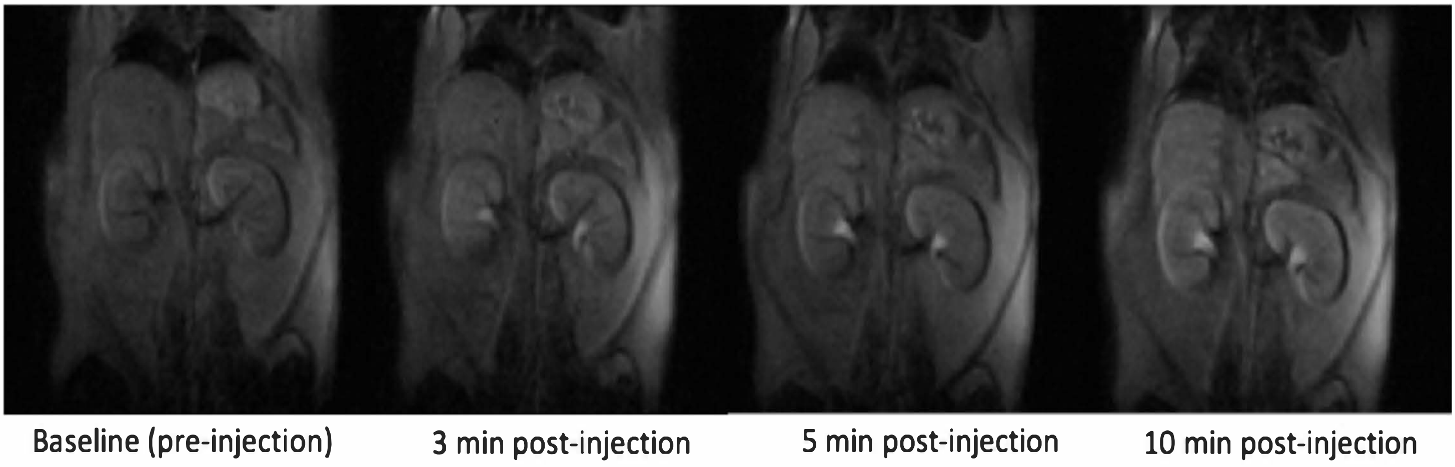

B

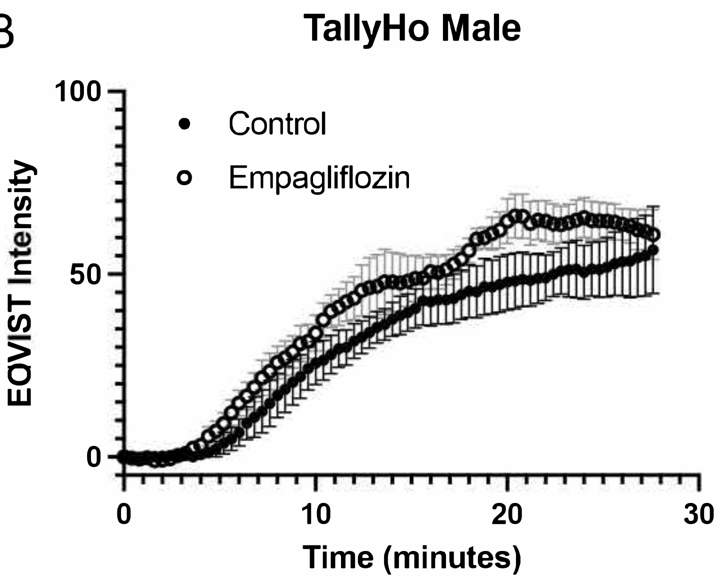

C

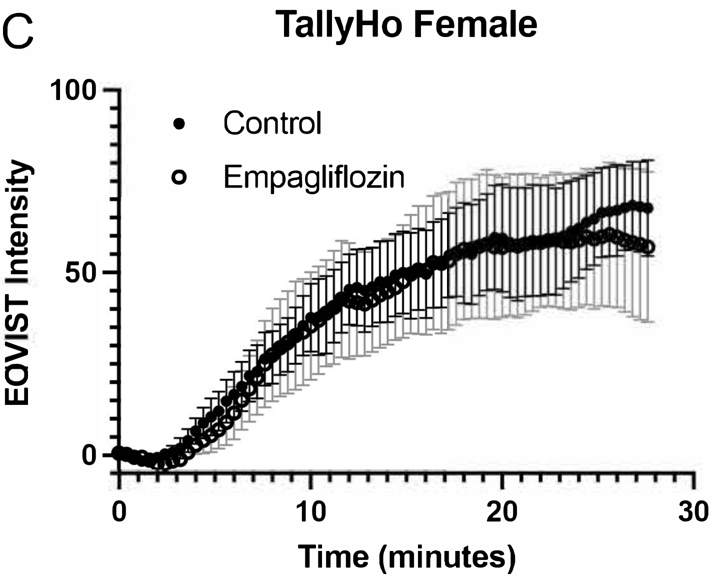

D

	Maximum Intensity	Time to peak intensity (minutes)	K_m (minutes)
Male Control	67.6 ± 12.7	22.9 ± 1.6	13.3 ± 0.6
Male Empagliflozin	88.8 ± 3.6	25.0 ± 1.5	12.8 ± 0.6
Female Control	95.0 ± 9.7	22.7 ± 2.4	13.4 ± 0.3
Female Empagliflozin	83.5± 23.7	24.7 ± 1.2	13.3 ± 0.5
