# Supplementary figures and images for: Empagliflozin Treatment Attenuates Hepatic Steatosis by Promoting White Adipose Expansion in Obese TallyHo Mice
Source: Int J Mol Sci. 2022 May 18;23(10):5675. doi: 10.3390/ijms23105675 (PMC9147974; doi:10.3390/ijms23105675)

Supplemental Figure S1

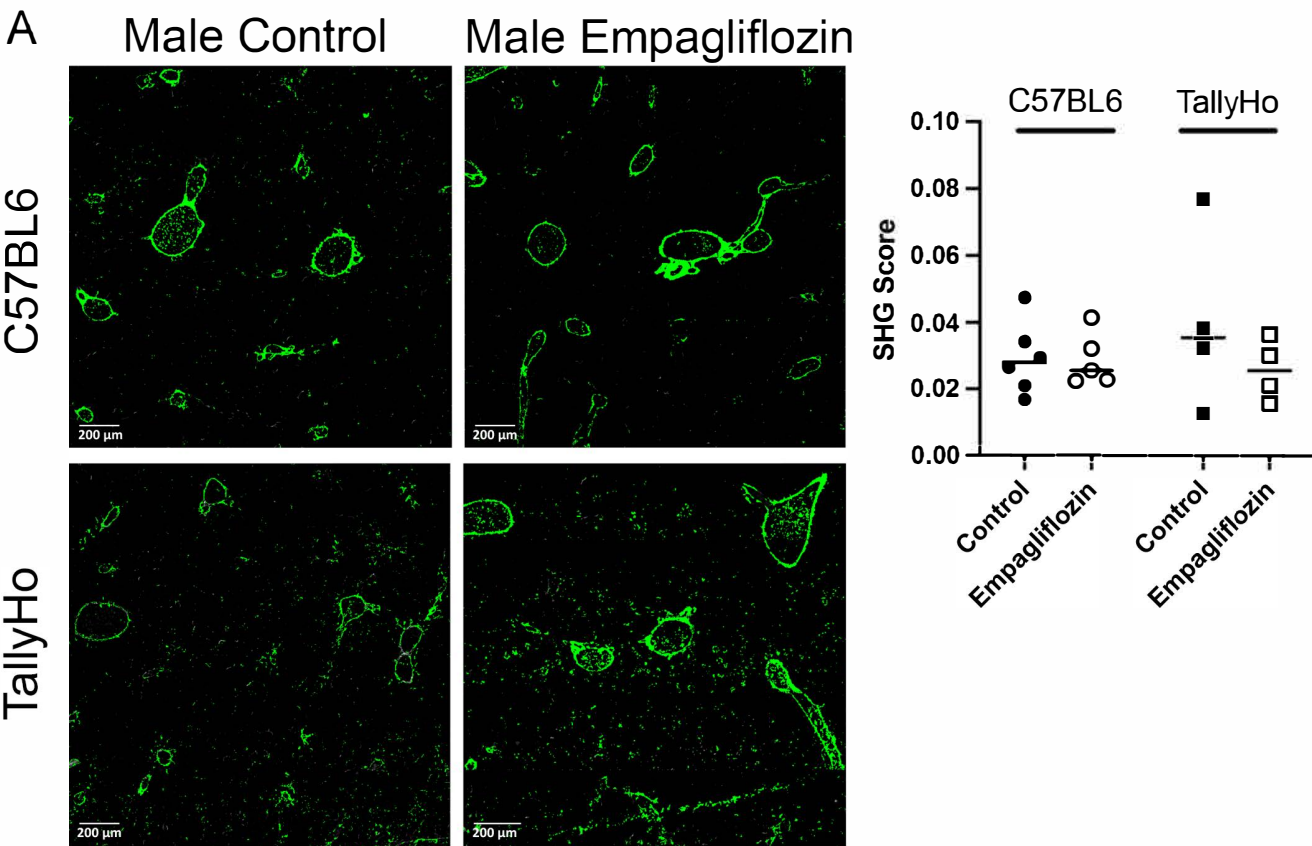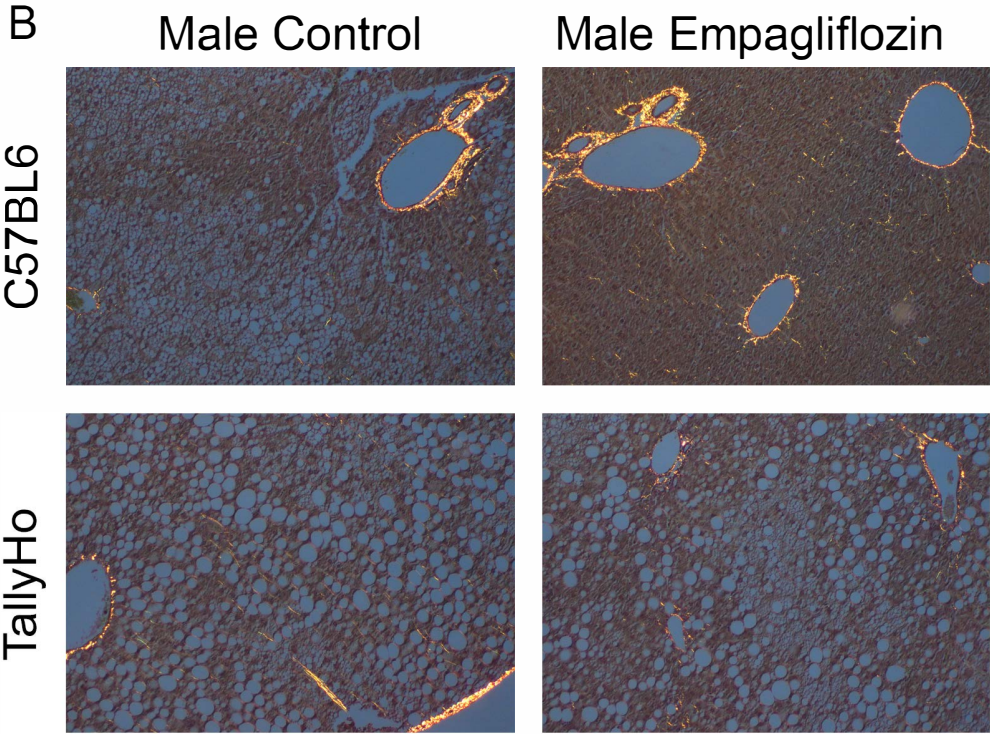

Supplement: Supplementary file 1 [file ijms-23-05675-s001.zip › Figure S1.pdf]

# Supplemental Figure S2

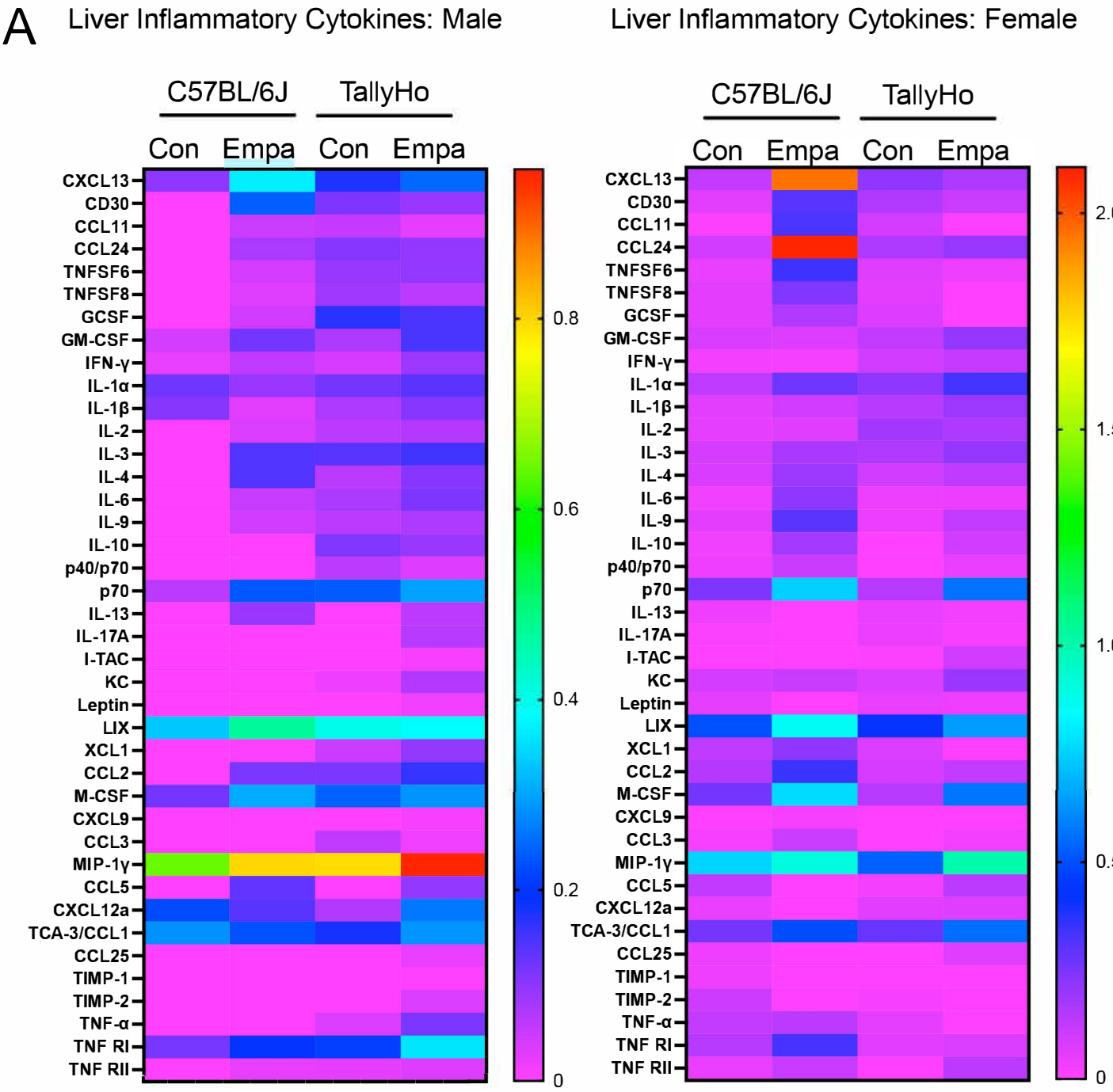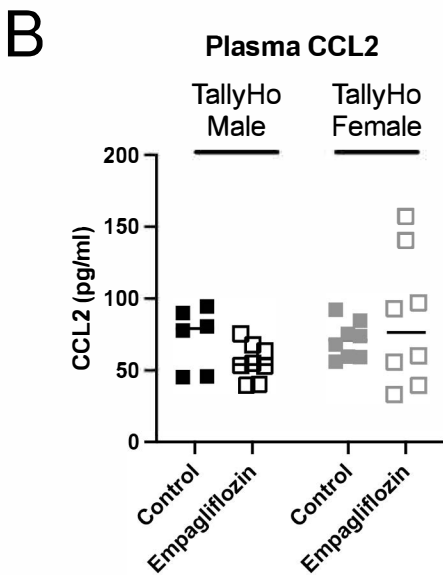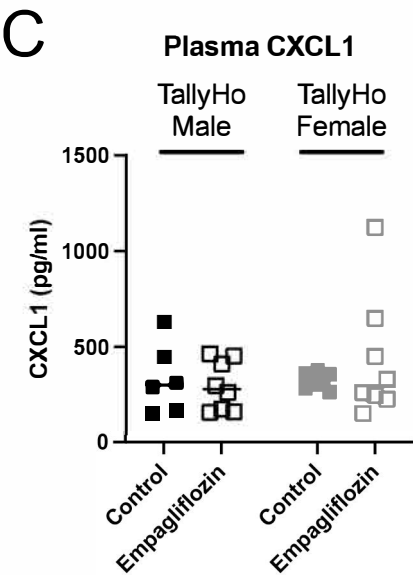

Supplement: Supplementary file 1 [file ijms-23-05675-s001.zip › Figure S2.pdf]
